# Enhanced Mechanical Properties and Anti–Inflammation of Poly(L–Lactic Acid) by Stereocomplexes of PLLA/PDLA and Surface–Modified Magnesium Hydroxide Nanoparticles

**DOI:** 10.3390/polym14183790

**Published:** 2022-09-10

**Authors:** Seung-Woon Baek, Jun Hyuk Kim, Duck Hyun Song, Da-Seul Kim, Chun Gwon Park, Dong Keun Han

**Affiliations:** 1 Department of Biomedical Science, CHA University, 335 Pangyo-ro, Bundang-gu, Seongnam-si 13488, Korea; 2 Department of Biomedical Engineering, SKKU Institute for Convergence, Sungkyunkwan University (SKKU), 2066 Seobu-ro, Jangan-gu, Suwon-si 16419, Korea; 3 Department of Intelligent Precision Healthcare Convergence, SKKU Institute for Convergence, Sungkyunkwan University (SKKU), 2066 Seobu-ro, Jangan-gu, Suwon-si 16419, Korea; 4 School of Integrative Engineering, Chung-Ang University, 84 Heukseok-ro, Dongjak-gu, Seoul 06974, Korea

**Keywords:** Poly(L–lactic acid), Poly(D–lactic acid), stereocomplex, magnesium hydroxide, biodegradable vascular scaffold, nanoparticles

## Abstract

Poly(L–lactic acid) (PLLA), as a biodegradable polymer, has attracted attention for use as a biomaterial. In order to apply PLLA as a cardiovascular stent, stronger mechanical properties and anti–inflammatory effects against acidic by–products are required. In this study, PLLA/PDLA stereocomplex microparticles (SC) were developed and surface–modified magnesium hydroxide (MH) nanoparticles with oligolactide were combined with these PLLA composites. The SC improved the mechanical properties of the PLLA composites through the formation of stereocomplex structures. The surface–modified MH nanoparticles showed enhanced mechanical properties due to the stereocomplex structures formed by PLLA chains and inhibited inflammatory responses by pH neutralization as a result of MH. Additionally, the MH nanoparticles containing PLLA composites had antibacterial effects and increased the viability of human vascular endothelial cells. This technology is expected to have great potential in the development of PLLA composite materials for the production of various medical devices, such as cardiovascular stents.

## 1. Introduction

Biodegradable polymers are extensively used in biomedical materials for tissue engineering and regeneration to facilitate the healing process [1,2,3]. Among them, poly(lactic acid) (PLA) has attracted attention as a biomaterial due to its non–toxicity, elasticity, biodegradability, good mechanical properties, and approval by the FDA. However, the production of certain medical devices, such as cardiovascular stents, requires biomaterials to have strong mechanical properties, and PLA is weaker than metal, resulting in recoil. Many researchers have devoted efforts to enhancing the mechanical properties of PLA. Wang et al. reported on the fabrication of tough PLA composites by adding poly(butylene adipate–co–terephthalate) (PBAT) and using a multifunctional epoxy oligomer as a crosslinker [4]. Deng et al. reported the development of PLA blends including ethylene–acrylic ester–glycidyl methacrylate terpolymers (EGMAs) via reactive blending and crystallization by annealing to obtain super–toughened PLA [5]. However, although the methods described above can improve the mechanical properties of PLA, they can also cause toxicity in the human body. Among the various methods for improving mechanical properties, the formation of stereocomplex structures is a method which allows for excellent biocompatibility. The stereocomplex structures formed by the mixing of enantiomeric poly(L–lactic acid) (PLLA) and poly(D–lactic acid) (PDLA) increase intermolecular interactions through the formation of hydrogen bonds and dipole–dipole interactions, resulting in tightly packed chains side by side, improving thermal stability, hydrolysis resistance, and mechanical properties [6,7,8]. Im et al. described the improvement of mechanical properties by in situ self–nucleated polymerization of PLLA using stereocomplex polylactide (SC–PLA) as a nucleating agent [9].

PLLA as polyester is degraded by hydrolysis in the body to produce lactic acid by–products. The lactic acid produced decreases local pH and induces inflammatory responses [10,11]. In our previous studies, the acidic by–products of biodegradable polymers were neutralized using magnesium hydroxide [Mg(OH)_2_, MH] [12,13,14]. MH is a biocompatible inorganic particle that has been widely used as an antacid agent. The pH–neutralizing effect of MH was found to reduce inflammatory response and improve tissue regeneration. In addition, MH has antibacterial properties [15,16]. Heydarian et al. reported that bacterial infection occurs the expression of pro–inflammatory cytokines, such as interleukin–6 (IL–6), interleukin–6 (IL–8), and tumor necrosis factor–α (TNF–α), in cells [17]. Unfortunately, the hydrophilic inorganic particles, such as MH, in hydrophobic polymers are aggregated and weaken the mechanical properties of the materials. To overcome these problems, many researchers have studied the surface modification of inorganic particles using ricinoleic acid, stearic acid, polylactic acid, and silane to improve interfacial bonding between PLLA and inorganic particles [18,19,20,21,22]. Previously, we have studied the surface modification of MH using monomers and oligomers of PLLA, such as DL–lactide, oligo(DL–lactic acid) (ODLLA), and oligo–D–lactide–ε–caprolactone (ODLCL) [23,24].

**Figure 1:**
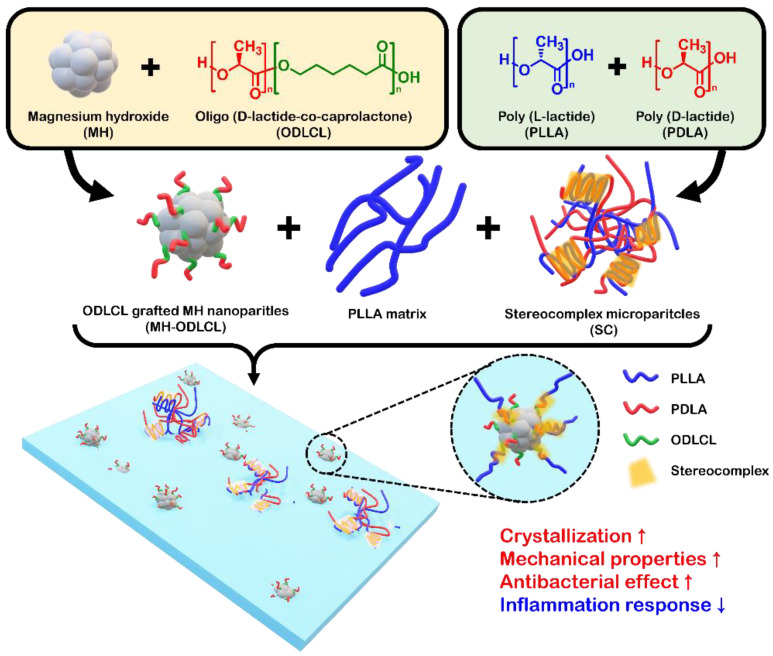
A schematic illustration of the preparation of the PLLA composites. The surface of MH was modified with ODLCL, and the SC were fabricated using PLLA and PDLA. The PLLA composites contained surface–modified MH and SC to provide anti–inflammatory effects and improved mechanical properties.

In this study, we fabricated PLLA composites including surface–modified MH nanoparticles and stereocomplex microparticles (SC) which had anti–inflammatory effects and improved mechanical properties. First, the SC, as homocomposites of PLLA and PDLA, were fabricated using an oil–in–water emulsion method. Secondly, based on the findings from our previous studies, the surface of MH was modified with oligo–D–lactide–ε–caprolactone (ODLCL) (MH–ODLCL). ODLCL, a copolymer of D–lactide and ε–caprolactone, forms a stereocomplex structure due to the domain of D–lactic acid in the matrix of PLLA. Finally, a composite composed of PLLA, SC, and MH–ODLCL was developed by the solvent casting method and hot–pressing for the reduction of inflammatory response and improved mechanical properties (Figure 1). After configuring the PLLA composites, the mechanical properties, degradation behavior, and expression of inflammatory factors were evaluated. These results indicated that the disadvantages of PLLA are mitigated and that the material can be applied in biodegradable vascular scaffolds.

## 2. Materials and Methods

### 2.1. Materials

Poly(L–lactic acid) (PLLA) and poly(D–lactic acid) (PDLA) were obtained from Samyang Biopharmaceutical Corp. (Seongnam-si, Korea). L–Lactide was obtained from DURECT Co. (Cupertino, CA, USA). D–Lactide was supplied by Haihang Industry Co., Ltd. (Jinan, Shandong, China). Magnesium hydroxide, ε–caprolactone, poly(vinyl alcohol) (PVA, Mw ≈ 13,000–23,000), tin(ΙΙ) 2–ethylhexananoate, and 1–octanol were obtained from Sigma–Aldrich (St. Louis, MO, USA). Toluene, chloroform, n–hexane, acetone, and dichloromethane (DCM) were supplied by Daejung Co. Ltd. (Seoul, Korea). IL–6 and IL–8 enzyme–linked immunosorbent assay (ELISA) kits were supplied by R&D Systems (Minneapolis, MN, USA). Phosphate–buffered saline (PBS) tablets were acquired from Thermo Fisher Scientific (Waltham, MS, USA). Proteinase K was obtained from Bioneer Co., Ltd. (Daejeon, Korea).

Gram–negative bacteria (*Escherichia coli* (*E. coli*)) and Gram–positive bacteria (*Staphylococcus saprophyticus* (*S saprophyticus*)) were supplied by the Korean Collection for Type Cultures (KCTC; Daejon, Korea). Luria–Bertani broth (LB broth), Luria–Bertani broth with agar (LB agar), nutrient broth, and nutrient agar were obtained from Sigma–Aldrich (St. Louis, MO, USA).

Human coronary artery endothelial cells (HCAECs) and EGM–2 media with an MV bullet kit were obtained from Lonza (Basel, Switzerland). A cell–counting kit (CCK–8) was purchased from Dongin LS (Korea). PBS solution was obtained from Hyclone (GE Healthcare Life Sciences, Logan, UT, USA). All chemicals were laboratory reagent grade and used without purification.

### 2.2. Preparation and Characterization of the Stereocomplex Microparticles (SC)

The SC were fabricated using a homogenization method. PLLA solution (DCM, 2 wt%) was added to 0.5 wt% aqueous PVA solution, followed by homogenization in a homogenizer operating at 10,000 rpm for 10 min (LSM–A, Silverson, Buckinghamshire, England). After that, the DCM was evaporated using an evaporator (N–1300, Eyela, Tokyo, Japan) for 3 h, and the SC were washed under centrifugation.

Field emission scanning electron microscopy (FE–SEM; S–4800, Hitachi, Tokyo, Japan) was used to observe the surface morphologies of the SC at 15 kv with the SE mode at 3.5 k. The samples were coated with platinum using an ion coater at 3 mA for 90 s. The average size and standard deviation of the SC were calculated from more than 500 particles in SEM images using ImageJ software (National Institutes of Health, Bethesda, MD, USA).

Differential scanning calorimetry (DSC; DSC4000, PerkinElmer, Waltham, MA, USA) was used to analyze the thermal behavior of the PLLA, PDLA, and their composites. Under nitrogen conditions, samples of approximately 5 mg were heated from 30 to 250 °C to measure non–isothermal crystallization. They were measured at a heating rate of 10 °C/min. The equation below was used to calculate the final crystallinity of homocrystallites and stereocomplex crystallites (*X_c_*_,*HC*_ and *X_c_*_,*SC*_, respectively):

$$X_{c}\left(\%\right)=\frac{\Delta H_{m}}{\Delta H_{f}w_{f}}\times 100$$

where Δ*H_m_* stands for the melting enthalpy for crystallization, ω_ƒ_ stands for the weight fraction of PLLA in the PLLA composites, and Δ*H_ƒ_* stands for the melting enthalpy for completely crystallized homocrystallites or stereocomplex crystallites (93 or 142 J/g). The equation below was used to calculate the proportion of stereocomplex crystallites (*ƒ_SC_*) [25]:

$$f_{SC}\left(\%\right)=\frac{X_{c,SC}}{X_{c,SC}+X_{c,HC}}\times 100\;\;$$

The crystalline structures of the PLLA, PDLA, and SC were examined using X-ray diffraction measurements. An X-ray diffractometer (XRD; D2 phaser, Bruker, Karlsruhe, Germany) and CuKα radiation was used to record the XRD patterns of the samples, and the degrees of crystallinity were estimated using crystalline peak areas. Profiles were recorded with a scattering angle range of 2θ = 0–30° and a scan speed of 0.05°/s [26,27].

An attenuated total reflection Fourier transform infrared spectrometer (ATR–FTIR; Spectrum Two FT–IR Spectrometer, Perkin Elmer, Waltham, MA, USA) with a resolution of 4 scans at 1 cm^−1^ and scales of 890–970 and 2920–3040 cm^−1^ was used to record the infrared spectra of the PLLA, PDLA, and SC.

### 2.3. Synthesis and Characterization of the Surface–Modified Magnesium Hydroxide

The ring–opening polymerization process was used to synthesize the OLLCL and ODLCL. Lactide and caprolactone were mixed in a ratio of 7:3 and then placed into a round beaker flask with toluene. After that, Tin(ΙΙ) 2–ethylhexananoate and 0.0453 mM of 1–octanol 3.839 mM were added, and the flask was purged with a nitrogen atmosphere. The polymerization occurred at 140 °C for 18 h. The synthesized oligomers were dissolved in chloroform, then precipitated in hexane to remove unreacted monomers and dried.

The oligomer–grafted MH was synthesized by combining the hydroxylate of MH and the carboxylate of the oligomers. The MH and oligomers (Mw ≈ 7 k) at a 1:1 ratio were placed into a round beaker flask with chloroform. After chloroform was evaporated at 90 °C for 6 h, the mixture was reacted under vacuum conditions at 150 °C for 15 h. The synthesized MH was washed using a co–solvent of chloroform/acetone (4:6) under centrifugation (7000 rpm, 10 min).

FE–SEM (15 kv, SE mode, ×130 k) and dynamic laser scattering (DLS; Zetasizer Nano ZS, Malvern Instruments, Worcestershire, UK) were used to observe surface morphologies and sizes. The samples were coated with platinum using an ion coater at 3 mA for 90 s. To observe dispersibility, the MH, MH–OLLCL, and MH–ODLCL were dispersed in chloroform and left for 3 h at room temperature. ATR–FTIR with a resolution of 4 scans at 1 cm^−1^ and a scale of 600–4000 cm^−1^ was used to record the infrared spectra. Thermogravimetric analysis (TGA; TGA 4000, PerkinElmer, Waltham, USA) was used to measure the grafting degree and composition. A mass–temperature curve was recorded under the test temperature range of 30–800 °C at a heating rate of 10 °C/min under nitrogen conditions.

### 2.4. Preparation and Characterization of the PLLA Composites

The PLLA composite manufacturing process was divided into two steps. In the first step, the solvent casting method was used to fabricate the PLLA composites. After 5 g of PLLA and 10 phr of oligomer–grafted MH were added to 70 mL of chloroform, the mixture was poured into a Teflon mold, and the solvent was evaporated at room temperature for 24 h. In the second step, a compression molding machine (QM900A, QMESYS, Gyangmyung, Korea) was used for the thermal melting and hot–pressing of the composites. The composites from the previous step and the SC (5 phr) were mixed and then hot–pressed at 160 °C for 5 min. FE–SEM (15 kv, SE mode, ×500) was used to observe the surface morphologies of the PLLA composites, and energy–dispersive spectroscopy (EDS) connected to the FE–SEM was used to measure the chemical compositions of the specimens. The samples were coated with platinum using an ion coater at 3 mA for 90 s. ATR–FTIR was used to record the infrared spectra of the PLLA composites, and TGA was used to measure the grafting degree and composition of the PLLA composites. The crystalline structures of the PLLA composites were examined via XRD. Profiles were recorded at a scattering angle range of 2θ = 0–40° and a scan speed of 0.05°/s. The thermal behavior of the PLLA composites was analyzed using a DSC.

### 2.5. Mechanical Properties

A universal testing machine (UTM; TO–101, Testone, Siheung, Korea) was used to investigate tensile strength, elongation, and Young’s modulus, following ASTM standard D638. The PLLA composites were produced as dumb–bell–shaped specimens (14 × 6 × 2 mm^3^). They were investigated at room temperature under a crosshead speed of 10 mm/min.

### 2.6. Degradation Behavior

To observe degradation behavior, the PLLA composites were fabricated into rectangular shapes (10 mm × 5 mm). After each sample was weighed, it was placed in 1 mL of PBS solution at pH 7.4 with proteinase K (0.04 mg/mL). This experiment was progressed at 37 °C for 4 days. A digital pH meter (Five Easy Plus, Mettler Toledo, Columbus, OH, USA) was used to evaluate the change in pH at identical times. To measure the remaining mass, a solution of the samples was washed with distilled water 3 times and dried under a vacuum. To measure the remaining mass, the solution of the samples was removed and dried under vacuum for 4 h. The following equation was used to calculate the weight loss of the samples [23]:

$$\mathrm{Weight}\;\mathrm{loss}\;\left(\%\right)=\frac{W_{AD}}{W_{BD\;\;}}\times 100$$

where *W_BD_* refers to the weight of the PLLA composites before degradation and *W_AD_* refers to the weight of the PLLA composites after degradation for a certain number of days.

### 2.7. Antibacterials Assay

*E. coli* were incubated in Luria–Bertani broth (LB broth; L3022, St. Louis, MO, USA, Sigma –Aldrich) and Luria–Bertani broth with agar (LB agar; L2897, St. Louis, MO, USA, Sigma–Aldrich) at 37 °C with aeration. *S. saprophyticus* were incubated in nutrient broth (70122, St. Louis, MO, USA, Sigma–Aldrich) and nutrient agar (70148, St. Louis, MO, USA, Sigma–Aldrich), also at 37 °C with aeration. After 16 h, the Gram–negative and Gram–positive bacteria were centrifuged and then resuspended in sterilized 0.85% NaCl solution. The density of the bacteria solution was calculated to be approximately 104 CFU (colony–forming units)/mL. The PLLA composites were added to 1 mL of the bacterial suspension for antibacterial testing. The composite scaffolds with the bacteria suspension were incubated at 37 °C for 1 day. Then, 100 μL of diluted bacterial suspension with the PLLA composites was spread on agar plates, and the plates were incubated overnight at 37 °C. The bactericidal effects were evaluated in terms of the CFUs using image J.

### 2.8. Cell Viability and Inflammation

HCAECs were grown in an EGM2–MV bullet kit in a humidified atmosphere with 5% carbon dioxide (CO_2_) at 37 °C. HCAECs were seeded at a density of 5 × 10^4^ cells/well in a 24–well cell culture plate and treated with degradation product at 60 °C for 21 days. After 24 h, the viability of cells and inflammatory response were determined using a CCK–8 assay kit and an ELISA kit for IL–6 and IL–8, respectively. The processes were conducted according to the provided protocols.

### 2.9. Statistical Analysis

The quantitative results were expressed as means ± standard deviations (SDs). # *p* < 0.0001, *** *p* < 0.001, ** *p* < 0.01, and * *p* < 0.05 indicate statistically significant differences. Statistical significance was evaluated by one–way analysis of variance (ANOVA) following Tukey’s method, using GraphPad Prism 7.0 software (GraphPad Software Inc., San Diego, CA, USA).

## 3. Results and Discussion

### 3.1. Characterization of the Stereocomplex Microparticles

**Figure 2:**
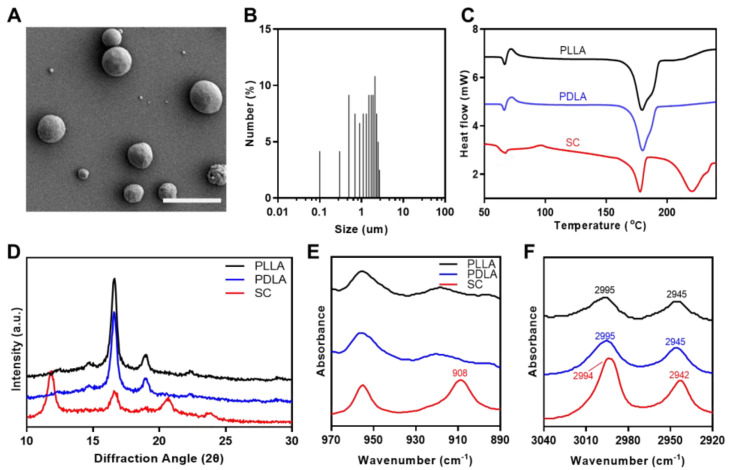
(**A**) SEM image and (**B**) size distribution of SC. Scale bar: 5 μm. (**C**) DSC thermograms, (**D**) XRD spectra, and (**E**,**F**) ATR–FTIR spectra of the PLLA, PDLA, and SC.

The stereocomplex microparticles (SC) were fabricated using a homogenizer to provide enhanced mechanical properties in a PLLA matrix. Figure 2A shows an SEM image of the SC. The SC exhibited spherical morphologies and were of various sizes. The average size of the SC measured using SEM imagery was 1.433 ± 0.704 μm (Figure 2B). Figure 2C displays DSC thermograms of a second heating scan of the PLLA, PDLA, and SC. The endothermic peak of homocrystallines of PLLA and PDLA were observed at 179 °C, whereas, for the SC observed the two endothermic homocrystallines and stereocomplex crystallines peaks at 178.21 and 220.28 °C, respectively [28,29,30]. Table 1 summarizes the corresponding parameters for the DSC thermograms for the PLLA, PDLA, and SC. These results showed a sharp decrease in *X*_*C,HC*_ and formation of *X*_*C,SC*_ and *f*_sc_ in the SC. Figure 2D shows XRD patterns for the PLLA, PDLA, and SC. The PLLA and PDLA had large homocrystal peaks at 2θ = 16.6 and 18.8°, while the SC showed large homocrystal peaks at 2θ = 16.7° as well as stereocomplex crystal peaks at 2θ = 11.8, 20.7, and 23.9° [27,31]. The FTIR spectra for the PLLA, PDLA, and SC are presented in Figure 2E and Figure 2F. As shown in Figure 2E, compared to the PLLA and PDLA, the SC showed FTIR absorption at 908 cm^−1^, indicating 3_1_–helical conformations of stereocomplex crystallines in the SC. Figure 2F displayed a low–frequency shift of C–H stretching at 2995 to 2994 cm^−1^ and 2945 to 2942 cm^−1^ by hydrogen bond formation between two groups among C–O, C–CH_3_, and C–CH_2_ stretching [25].

### 3.2. Characterization of the Surface–Modified Magnesium Hydroxide Nanoparticles

**Figure 3:**
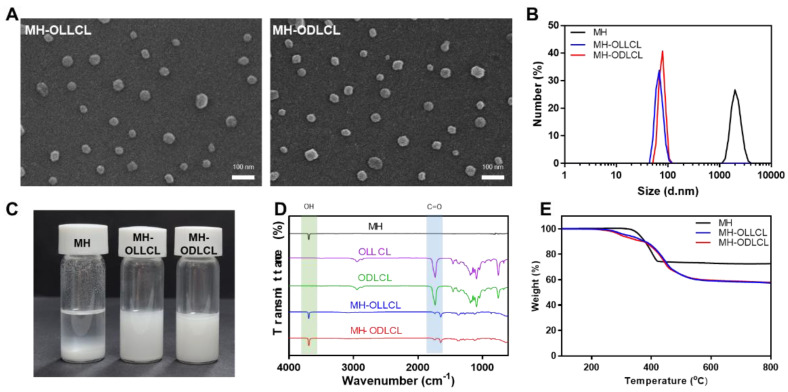
Characterization of the surface–modified MHs. (**A**) SEM images of the MH–OLLCL and MH–ODLCL. Scale bar: 100 nm. (**B**) Size distribution, (**C**) optical image of dispersion in an organic solvent, (**D**) ATR–FTIR spectra, and (**E**) TGA thermograms of the MHs, MH–OLLCL, and MH–ODLCL.

Figure 3A shows an SEM image of the surface–modified MH nanoparticles (MH–OLLCL and MH–ODLCL). The sizes of both the MH–OLLCL and MH–ODLCL were approximately 40 nm. As shown in Figure 3B, the average sizes of the MH, MH–OLLCL, and MH–ODLCL particles in an organic solvent analyzed by DLS were 2111 ± 449.8, 75.56 ± 13.24, and 76.93 ± 10.25 nm, respectively, which were slightly larger than the sizes determined from the SEM images due to the hydrodynamic volumes of the DLS method. The sizes of the MH–OLLCL and MH–ODLCL were smaller than those of the MHs, because the hydrophilic MHs aggregated in the organic solvent, whereas the surface–modified MH–OLLCL and MH–ODLCL with their hydrophobic oligomers had high stabilities in the organic solvent. Figure 3C shows the dispersion of the MHs, MH–OLLCL, and MH–ODLCL. Compared to the MHs, the surface–modified MH nanoparticles showed stable dispersity. The chemical structures of the synthesized MH–OLLCL and MH–ODLCL were analyzed by ATR–FTIR (Figure 3D). The unmodified MHs had peaks of –OH stretching at 3699 cm^−1^. The peaks at 1760 cm^−1^ of the oligomers (OLLCL and ODLCL) represented the carbonyls of the ester groups. The MH–OLLCL and MH–ODLCL had peaks of shifted ester bonds at 1644 cm^−1^, which demonstrated that the OLLCL and ODLCL had been successfully modified on the surface of the MH nanoparticles. Figure 3E shows the amounts of OLLCL and ODLCL grafted on the surface of MHs measured by TGA. The weights of unmodified MH nanoparticles decreased in the temperature range of 280 to 450 °C, as the MH decomposed to magnesium oxide (MgO). The weight loss for the MHs reached 32.7% at 700 °C, and the weight losses for the MH–OLLCL and MH–ODLCL were 42.6 and 42.2%, respectively [32]. When the remaining MgO was converted into MH, the amounts of MH–OLLCL and MH–ODLCL were 16.9 and 16.3%, respectively.

### 3.3. Characterization of the PLLA Composites

**Figure 4:**
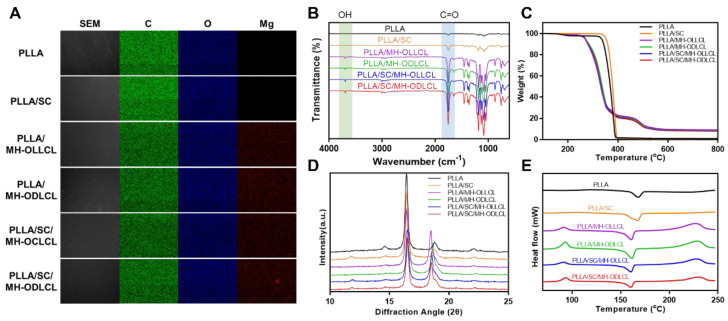
Characterization of the PLLA composites. (**A**) SEM image, (**B**) ATR–FTIR spectra, (**C**) TGA thermograms, (**D**) XRD spectra, and (**E**) DSC thermograms of the PLLA, PLLA/SC, PLLA/MH–OLLCL, PLLA/MH–ODLCL, PLLA/SC/MH–OLLCL, and PLLA/SC/MH–ODLCL composites.

Figure 4A shows SEM images and corresponding EDS mappings for the PLLA, PLLA/SC, PLLA/MH–OLLCL, PLLA/MH–ODLCL, PLLA/SC/MH–OLLCL, and PLLA/SC/MH–ODLCL nanoparticles. All samples displayed smooth and uniform surfaces. The distribution of C, O, and Mg elements in each sample was observed via EDX analysis. The C and O mapping results were similar across all samples. While Mg elements were not detected in the PLLA and PLLA/SC nanoparticles, they were evenly distributed in the PLLA/MH–OLLCL, PLLA/MH–ODLCL, PLLA/SC/MH–OLLCL, and PLLA/SC/MH–ODLCL composites. To further confirm the MH in the PLLA composites, the PLLA, PLLA/SC, PLLA/MH–OLLCL, PLLA/MH–ODLCL, PLLA/SC/MH–OLLCL, and PLLA/SC/MH–ODLCL nanoparticles were measured by ATR–FTIR (Figure 4B). Compared with the PLLA and PLLA/SC composites, the PLLA/MH–OLLCL, PLLA/MH–ODLCL, PLLA/SC/MH–OLLCL, and PLLA/SC/MH–ODLCL nanoparticles had an –OH stretching absorption peak for MH at 3697 cm^−1^. The amounts of MH contained in the PLLA composites were measured using TGA thermograms (Figure 4C). The PLLA/MH–OLLCL, PLLA/MH–ODLCL, PLLA/SC/MH–OLLCL, and PLLA/SC/MH–ODLCL composites contained approximately 8% MH. In Figure 4D, to investigate crystallization by the stereocomplex structures, XRD patterns were estimated. The PLLA/SC, PLLA/SC/MH–OLLCL, and PLLA/SC/MH–ODLCL composites containing SC showed weak stereocomplex crystal peaks at 2θ = 11.8 [27,31]. However, a stereocomplex crystal peak was not observed for the PLLA/MH–ODLCL because it was hidden by the strong crystal peak of MH. To further analyze stereocomplex structures, the nonisothermal crystallization and melting behaviors of PLA composites were evaluated by DSC (Figure 4E). Table 2 lists the thermal parameters obtained for the PLLA composites. All samples had T_g_ ranges from 55 to 63 °C and a T_m_ for homocrystallines of approximately 166 °C. The PLLA/MH–OLLCL, PLLA/MH–ODLCL, PLLA/SC/MH–OLLCL, and PLLA/SC/MH–ODLCL composites containing MH indicated exothermic peaks at the T_m_ position for stereocomplex crystallines, since the PLLA/MH showed an exothermic peak at about 230 °C (Figure S1). However, it had a different melting enthalpy by offsetting between the exothermic peak of MH and the endothermic peak of stereocomplex crystallines. The melting enthalpies of the PLLA/MH–OLLCL, PLLA/MH–ODLCL, PLLA/SC/MH–OLLCL, and PLLA/SC/MH–ODLCL composites were 66.00, 52.49, 32.65, and 32.04 J/g, respectively. The MH–ODLCL had lower melting enthalpies than the MH–OLLCL due to their stereocomplex structures. Compared with the MH–OLLCL and MH–ODLCL, the melting enthalpies of the PLLA/SC/MH–OLLCL and PLLA/SC/MH–ODLCL composites were greatly reduced by the SC. The stereocomplex crystal peak for the PLLA/SC nanoparticles was also not exhibited. Tashiro et al. investigated stereocomplex crystal peaks according to PLLA/PDLA blend samples with various L/D form ratios [33]. They demonstrated that the stereocomplex crystal peak did not appear below a 9/1 ratio. Figure S2 shows ATR–FTIR spectra for the PLLA and PLLA/SC particles which demonstrate the stereocomplex structures of the PLLA/SC nanoparticles. The PLLA/SC showed FTIR absorption at 908 cm^−1^, indicating 3_1_–helical conformations of stereocomplex crystallines [25]. These results suggest that stereocomplex structures were formed in the PLLA composites by the SC and MH–ODLCL.

### 3.4. Mechanical Properties of the PLLA Composites

**Figure 5:**
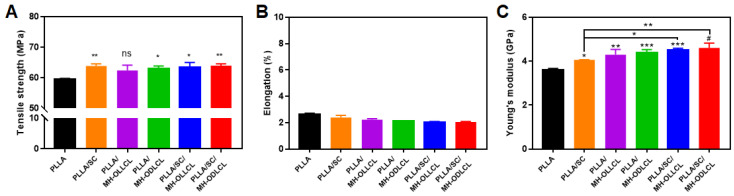
Mechanical properties of the PLLA, PLLA/SC, PLLA/MH–OLLCL, PLLA/MH–ODLCL, PLLA/SC/MH–OLLCL, and PLLA/SC/MH–ODLCL composites: (**A**) tensile strength, (**B**) elongation, and (**C**) Young’s modulus (* p < 0.05, ** *p* < 0.01, *** *p* < 0.001, # *p* < 0.0001).

The mechanical properties of the PLLA composites were investigated by UTM. Figure 5A shows the tensile strengths of the PLLA composites. The PLLA/MH–OLLCL composite had a similar result to pure PLLA. In contrast, the tensile strength of the PLLA/MH–ODLCL composite increased compared to that of pure PLLA, because the MH–ODLCL formed stereocomplex structures with PLLA chains. The PLLA/SC, PLLA/SC/MH–OLLCL, and PLLA/SC/MH–ODLCL composites, in which stereocomplex structures formed between the SC and PLLA chains, had significantly improved tensile strengths of 63.7, 63.3, and 64.0 MPa, respectively. The elongation of all samples was around 2% (Figure 5B). Surface–modified MH and SC in the PLLA composites did not affect elongation. The Young’s moduli for the PLLA, PLLA/SC, PLLA/MH–OLLCL, PLLA/MH–ODLCL, PLLA/SC/MH–OLLCL, and PLLA/SC/MH–ODLCL composites were 4.06, 4.30, 4.38, 4.50, and 4.61 GPa, respectively. The inclusion of SC increased the Young’s moduli by the formation of stereocomplex structures. The inclusion of surface–modified MH increased interaction between the PLLA chains and the surface–modified MH, resulting in improved Young’s moduli. Among the PLLA composites containing surface–modified MH, the PLLA/MH–ODLCL composite showed an enhanced Young’s modulus relative to the PLLA/MH–OLLCL composite due to the formation of stereocomplex structures. When both the surface–modified MH and SC were included, the Young’s modulus increased further as a result of their synergistic effect, and the PLLA/SC/MH–ODLCL composite had the highest Young’s modulus.

### 3.5. Degradation Behavior of the PLLA Composites

**Figure 6:**
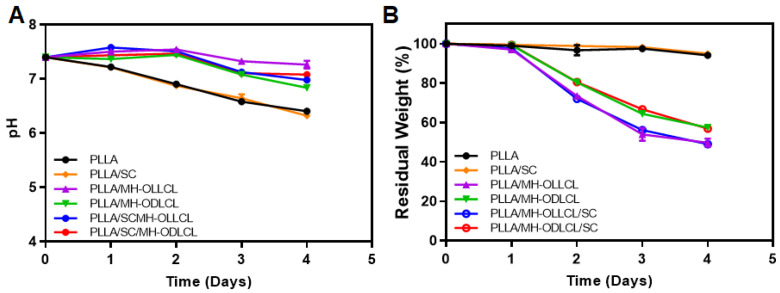
Changes in (**A**) pH value and (**B**) residual weight during degradation in PBS solution with proteinase K at 37 °C over 4 days for the PLLA, PLLA/SC, PLLA/MH–OLLCL, PLLA/MH–ODLCL, PLLA/SC/MH–OLLCL, and PLLA/SC/MH–ODLCL composites.

The degradation of the PLLA composites took place under accelerated conditions with proteinase K in PBS solution over 4 days. Figure 6A shows the change in pH values for the PLLA, PLLA/SC, PLLA/MH–OLLCL, PLLA/MH–ODLCL, PLLA/SC/MH–OLLCL, and PLLA/SC/MH–ODLCL composites. The pH values for the PLLA and PLLA/SC continuously decreased during degradation over the 4 days to 6.4 and 6.3, respectively. Meanwhile, the PLLA/MH–OLLCL, PLLA/MH–ODLCL, PLLA/SC/MH–OLLCL, and PLLA/SC/MH–ODLCL composites maintained neutral pHs at 7.22, 6.89, 6.98, and 7.08, respectively, because the MH in the PLLA composites released basic magnesium ions that neutralized the acidic by–products of the PLLA [34]. The residual weights of the PLLA and PLLA/SC did not degrade and were maintained at around 100% over the 4 days. In the PLLA/MH–OLLCL and PLLA/SC/MH–OLLCL composites, the residual weights decreased to 49.73 and 49.23%, respectively, over the 4 days. These results were due to the accelerated degradation, as MH was initially released and water molecules penetrated the MH–released sites. Compared to the PLLA composites containing MH–OLLCL, the PLLA/MH–ODLCL and PLLA/SC/MH–ODLCL composites were slowly degraded due to their being densely packed and their enhanced intermolecular interactions (57.43 and 58.89%, respectively). Thus, the PLLA composites with MH can biodegrade within 24 months to suppress the inflammation that occurs during long–term implantation.

### 3.6. Biological Properties of the PLLA Composites

**Figure 7:**
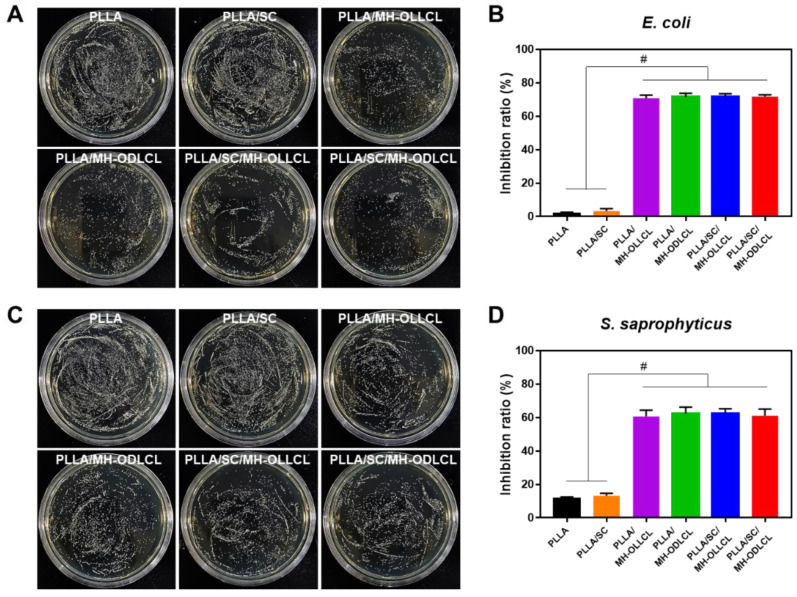
The bactericidal activity of each PLLA composite was investigated. Representative images showing the antimicrobial activities against Gram–negative bacteria (*E. coli*; **A**,**B**) and Gram–positive bacteria (*S. saprophyticus*; **C**,**D**) of the PLLA and the PLLA/SC, PLLA/MH–OLLCL, PLLA/MH–ODLCL, PLLA/SC/MH–OLLCL, and PLLA/SC/MH–ODLCL composites and their respective quantifications (*# p* < 0.0001).

Figure 7 shows results of the investigation of the bactericidal activity of each of the PLLA composites. Antimicrobial activity results for the PLLA and the PLLA/SC, PLLA/MH–OLLCL, PLLA/MH–ODLCL, PLLA/SC/MH–OLLCL, and PLLA/SC/MH–ODLC composites showed that the Gram–negative bacteria (*E. coli*) were inhibited by 1.72, 3.32, 70.86, 71.66, 71.75, and 71.80%, respectively, and that the Gram–positive bacteria (*S. saprophyticus*) were inhibited by 11.71, 13.33, 60.62, 62.28, 62.35, and 61.07%, respectively. Compared to the PLLA and the PLLA/SC, the PLLA composites containing MH significantly inhibited both *E. coli* and *S. saprophyticus* due to the presence of MH. These results suggest that the PLLA composites containing MH have antibacterial effects, since the adsorption of Mg^2+^ ions destroys bacterial cell walls and induces cell death [16,35,36,37,38,39].

The cell viability of HCAECs was evaluated under treatment with L–lactic acid (L–Lac, 12 mM), the degradation product of PLLA, and with surface–modified MH (Figure S3). The cell viability of HCAECs was reduced to 36% when treated with L–Lac. On the other hand, the L–Lac and surface–modified MH treated groups demonstrated cell viabilities of more than 80%. On the basis of these results, it was demonstrated that the acidic degradation products of PLLA induced damage to HCAECs and that the MH inhibited damage to HCAECs by neutralizing the acidic degradation products.

**Figure 8:**
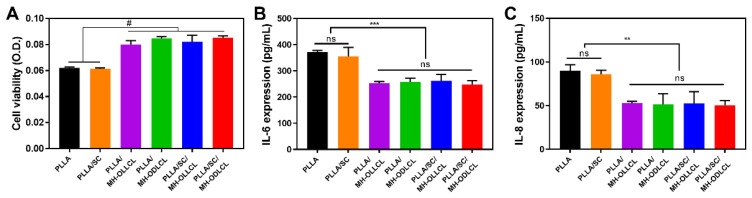
In vitro biocompatibility test of the PLLA and PLLA/MH, PLLA/SC, PLLA/MH–OLLCL, PLLA/MH–ODLCL, PLLA/SC/MH–OLLCL, and PLLA/SC/MH–ODLCL composites. (**A**) HCAECs cell viability with the degradation product of the PLLA composites treatment for 24 h. The expressions of (**B**) IL–6 and (**C**) IL–8 in HCAECs after 24 h, as determined by ELISA (** p < 0.01, *** *p* < 0.001, # *p* < 0.0001).

To investigate the biocompatibility of the PLLA composites in vitro, HCAECs were treated with degradation products obtained under accelerated conditions (Figure 8). Figure 8A shows cell viability at 24 h as quantified by CCK–8. Compared to the PLLA/MH–OLLCL, PLLA/MH–ODLCL, PLLA/SC/MH–OLLCL, and PLLA/SC/MH–ODLCL composites, the cell viability of the PLLA and PLLA/SC decreased due to acidic degradation by–products. On the other hand, cell viability did not decrease for the PLLA composites including MH due to the neutralization of the acidic degradation products by MH. In addition, the release of magnesium ions by dissociated MH affects mitochondrial RNA splicing protein 2 (MRS2) and transient receptor potential cation channel subfamily M member 7 (TRPM7) and maintains the homeostasis of HCAECs [10]. The expression of pro–inflammatory cytokines was investigated using an ELISA kit (Figure 8B,C). Compared to PLLA and PLLA/SC, the PLLA/MH–OLLCL, PLLA/MH–ODLCL, PLLA/SC/MH–OLLCL, and PLLA/SC/MH–ODLCL composites showed decreased expression of IL–6 and IL–8. Consequently, MH significantly prevented noninfectious inflammatory reactions caused by lactic acid derived from PLLA. Riemann et al. reported that an acidic environment leads to pathological gene expression and induces an inflammatory response [34,40].

## 4. Conclusions

Among biodegradable polymers, PLLA has attracted attention as a biomaterial due to its non–toxicity, elasticity, biodegradability, and good mechanical strength. However, it is unsuitable for producing items that require high mechanical strength, such as cardiovascular stents, and pH at the implant site in the body is reduced by its degradation products, which induces an inflammatory response. In this study, the composite, including SC and modified MH in PLLA, was successfully prepared for formation of sterocomplex structure and anti-inflammatory effect.

We investigated the formation of stereocomplex structures and the anti–inflammatory effects of SC and surface–modified MH in the PLLA composites. The PLLA composites containing SC and surface–modified MH showed significantly improved mechanical properties due to the existence of stereocomplex structures and inhibited inflammatory responses in vascular endothelial cells. In addition, they recently displayed antibacterial effects that are considered important in biomedical materials [39,41].

There is a limitation to this study. Since it is known that the degradation period of PLLA is more than 24 months, we measured the degradation behavior of PLLA composites using proteinase K under accelerated conditions [42]. When the PLLA was hydrolyzed at 37 °C, the pH value remained neutral for up to 40 weeks and the mechanical strength decreased from 6 months [42,43]. Therefore, it can be inferred that the method using proteinase K quickly passed the period of maintained mechanical strength. In a future study, we plan to degrade the PLLA composites at 37 °C over 2 years to measure their mechanical properties and degradation behavior.

These PLLA composites are expected to be applied in the future as biomaterials in the production of such items as biodegradable vascular scaffolds with improved mechanical properties and reduced cytotoxicity.

## Figures and Tables

**Table 1:** Thermal properties of the PLLA, PDLA, and SC.

	T_g_ (°C)	T_c_ (°C)	T_m,HC_ (°C)	ΔH_m,HC_ (J/g)	T_m,SC_ (°C)	ΔH_m,SC_ (J/g)	*X_C,HC_* (%)	*X_C,SC_* (%)	*f_SC_* (%)
PLLA	66.68	111.92	179.42	64.49	–	–	69.34	–	–
PDLA	66.62	104.62	179.88	63.08	–	–	67.83	–	–
SC	67.76	112.18	178.21	15.97	220.28	28.84	17.17	20.31	54.19

**Table 2:** Thermal properties of the PLLA, PLLA/SC, PLLA/MH–OLLCL, PLLA/MH–ODLCL, PLLA/SC/MH–OLLCL, and PLLA/SC/MH–ODLCL composites.

	T_g_ (°C)	T_c_ (°C)	T_m,HC_ (°C)	ΔH_m,HC_ (J/g)	*X_C,HC_* (%)
PLLA	63.37	–	167.03	8.971	9.64
PLLA/SC	55.98	111.98	167.47	28.19	30.31
PLLA/MH–OLLCL	56.51	91.78	160.58	32.46	34.90
PLLA/MH–ODLCL	56.93	93.18	161.38	30.29	32.56
PLLA/SC/MH–OLLCL	55.81	91.32	160.45	27.95	30.05
PLLA/SC/MH–ODLCL	56.32	93.79	160.55	27.45	29.51

## Data Availability

The data used in this study are presented in the text. Additional information can be made available upon request from the corresponding author.

## Supplementary Materials

The following supporting information can be downloaded at: https://www.mdpi.com/article/10.3390/polym14183790/s1, Figure S1: DSC thermograms of the PLLA/MH, Figure S2: ATR–FTIR spectra of the PLLA and PLLA/SC, Figure S3: Cell viability of HCAEC in 12 mM L–Lac with surface–modified MH at a concentration of 10 phr for 24 h.
