# Supplementary material for: Enhanced Mechanical Properties and Anti–Inflammation of Poly(L–Lactic Acid) by Stereocomplexes of PLLA/PDLA and Surface–Modified Magnesium Hydroxide Nanoparticles
Source: Polymers (Basel). 2022 Sep 10;14(18):3790. doi: 10.3390/polym14183790 (PMC9504497; doi:10.3390/polym14183790)
Supplement: Supplementary file 1 [file polymers-14-03790-s001.zip › polymers-1893001-supplementary.pdf]

Supplementary Information

Enhanced Mechanical Properties and Anti–Inflammation of Poly(L–Lactic Acid) by Stereocomplexes of PLLA/PDLA and Surface–Modified Magnesium Hydroxide Nanoparticles

Seung-Woon Baek [1,2,3,†], Jun Hyuk Kim [1,†], Duck Hyun Song [1], Da-Seul Kim [1,4], Chun Gwon Park [2,3,*] and Dong Keun Han [1,*]

1 Department of Biomedical Science, CHA University, 335 Pangyo-ro, Bundang-gu, Seongnam-si 13488, Korea
2 Department of Biomedical Engineering, SKKU Institute for Convergence, Sungkyunkwan University (SKKU), 2066 Seobu-ro, Jangan-gu, Suwon-si 16419, Korea
3 Department of Intelligent Precision Healthcare Convergence, SKKU Institute for Convergence, Sungkyunkwan University (SKKU), 2066 Seobu-ro, Jangan-gu, Suwon-si 16419, Korea
4 School of Integrative Engineering, Chung-Ang University, 84 Heukseok-ro, Dongjak-gu, Seoul 06974, Korea
* Correspondence: chunpark@skku.edu (C.G.P.); dkhan@cha.ac.kr (D.K.H.)
† These authors contributed equally to this work.

.

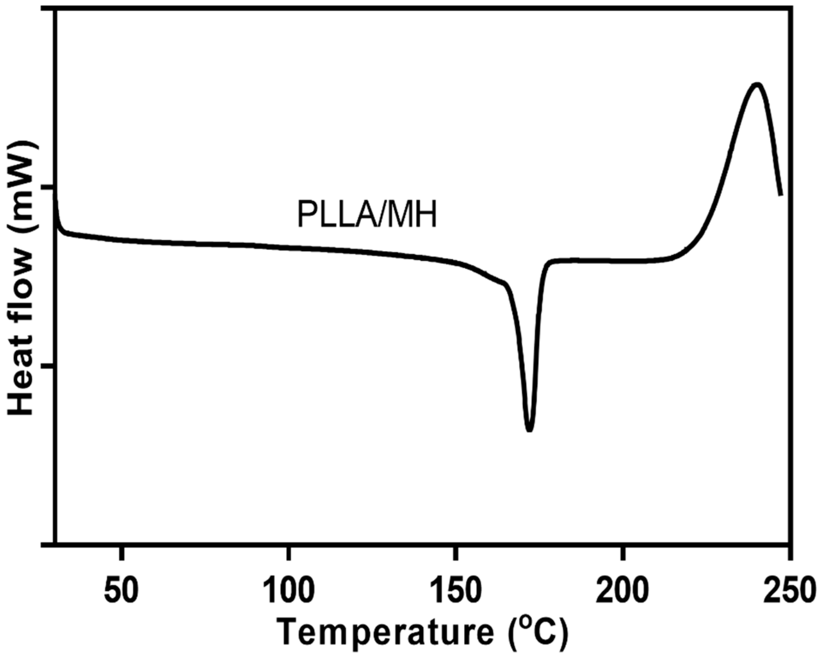

Figure S1. DSC thermogram of the PLLA/MH.

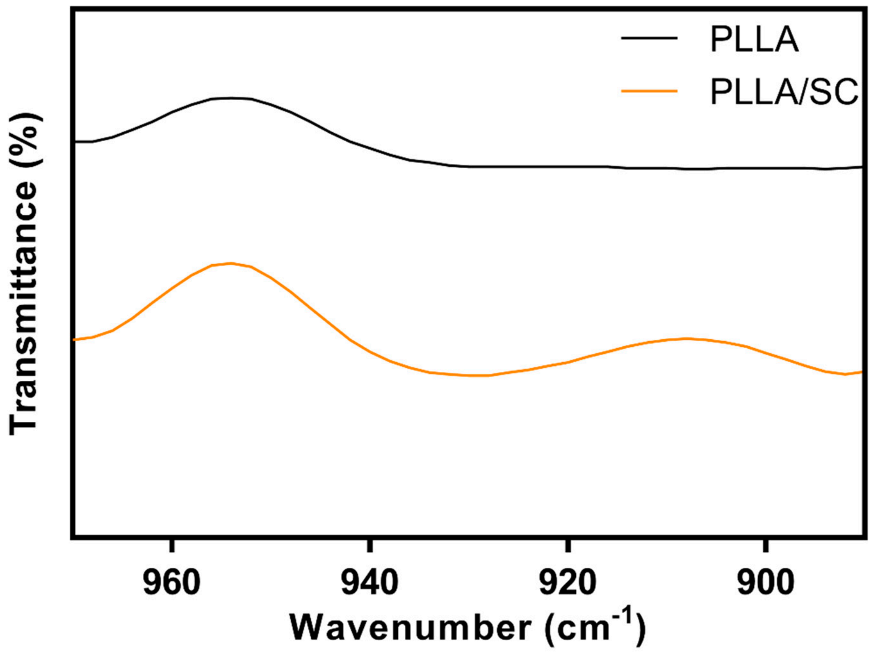

Figure S2. ATR-FTIR spectra of the PLLA and PLLA/SC.

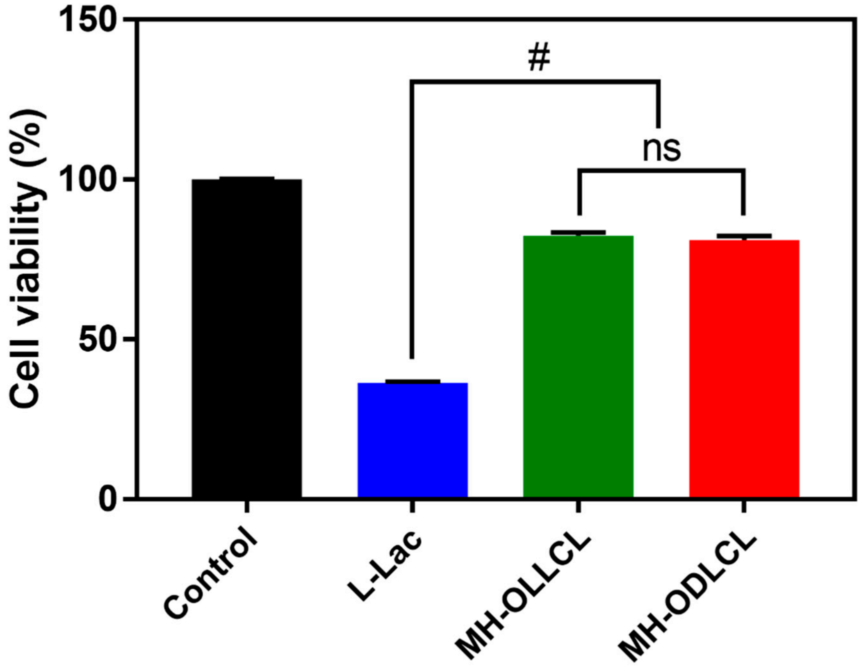

Figure S3. Cell viability of HCAEC in 12 mM L-Lac with surface-modified MH at a concentration of 10 phr for 24 h (**p*<0.05, ***p*<0.01, ****p*<0.001, #*p*<0.0001).
